# Reproductive characteristics of the hermaphroditic four-finger threadfin, *Eleutheronema tetradactylum* (Shaw, 1804), in tropical coastal waters

**DOI:** 10.1186/s40850-023-00181-w

**Published:** 2023-09-19

**Authors:** Kay Khine Soe, Teuku Haris Iqbal, Apiradee Lim, Wen‑Xiong Wang, Karl W. K. Tsim, Yutaka Takeuchi, Nirattisai Petchsupa, Sukree Hajisamae

**Affiliations:** 1 Faculty of Science and Technology, Prince of Songkla University, Muang, Pattani, 94000 Thailand; 2 Department of Fisheries Resources Utilization, Faculty of Marine and Fisheries, Universitas Syiah Kuala, Banda Aceh, 23111 Indonesia; 3 School of Energy and Environment and State Key Laboratory of Marine Pollution, City University of Hong Kong, Kowloon, Hong Kong, China; 4 Division of Life Science, Hong Kong University of Science and Technology, Clear Water Bay, Kowloon, Hong Kong, China; 5 Noto Center for Fisheries Science and Technology, Faculty of Biological Science and Technology, Kanazawa University, Ossaka, Noto-Cho, Ishikawa Japan

**Keywords:** Fecundity, Gonadosomatic index, Size at first maturity, Size and weight at first sex change, Spawning season

## Abstract

This study investigated the reproductive traits of the hermaphroditic four-finger threadfin, *Eleutheronema tetradactylum*, along the coasts of Thailand during January to December 2021. Fish samples were collected from Pattani Bay, Thailand to assess the sex ratio, gonadosomatic index (GSI), maturity stage and fecundity. Additional fish samples were also collected from other areas to evaluate the length and weight at first sex change (Ls_50_ and Ws_50_) and length at first maturity (Lm_50_). The overall sex ratio for male and female was 1:0.69 with male being predominant throughout the year. Threadfin fish spawn the whole year round with peaks during moderate rainy and heavy rainy seasons. Histological examination confirmed its protandrous hermaphrodite posing multiple spawning habits. The average fecundity was 1.85 × 10^5^ ± 1.05 × 10^5^ eggs and positively related with standard length, body weight, gonad weight, and egg diameter (*p* < 0.05). The Ls_50_ and Ws_50_ were 27.58 cm and 419.39 g, and 29.71 cm and 457.28 g, for fish from Pattani Bay and Samut Prakan province, respectively. The Lm_50_ of male from Pattani Bay and Samut Prakan province were 25.78 cm and 25.56 cm, respectively, which were larger than those from Satun and Nakhon Sri Thammarat provinces. The Lm_50_ of females from Pattani Bay was smaller than that from Samut Prakan province. This study provided fundamental information on the reproductive characteristics of *E. tetradactylum*, which can be implemented to support management of natural fish stock and aquaculture development.

## Introduction

Information on fish reproductive biology is crucial for managing the fishery resources and aquaculture [1, 2]. Several parameters such as sex ratio, gonadosomatic index (GSI), maturity stage, fecundity, size and weight at first sex change, size at first maturity and relationship between various parameters with reproductive traits have been reported in many fish species, e.g., neotropical fish; *Astyanax fasciatus*, *Oligosarcus robustus*, *Loricariichthys anus*, *Trachelyopterus lucenai* [1] and other 1207 marine fish species from Brazil [2]. The four-finger threadfin, *Eleutheronema tetradactylum*, is widely distributed along the coasts of India, Bangladesh, Sri Lanka, the Indo-West Pacific and the northern and western Australia [3–5]. Its habitats range from coastal waters to estuaries in the tropics [5–8]. It has long been a target fish species by fishermen in many parts of the world, including Thailand, with high value and market demand [9]. The fishing gears used typically to capture this fish include gillnet, beach seine, longline, trap and trawl [10].

Many studies were conducted on the biology and fisheries of this species including its breeding [8], length at maturity size [11], fecundity [12], histology of gonads [13], stock structure and exploitation status [10, 14, 15], aquaculture [16], taxonomy [3–5] and compositions of parasitic organisms [17, 18]. However, most studies on reproductive aspects focused only on a particular reproductive trait viz., length and weight relationship [10, 11, 14], fecundity [8, 12], gonad histology [13], size at first maturity [10], and size at the first sex change [14]. Additionally, some studies reported that this fish was found from late winter to mid-summer in Australia [7], March to September in Malaysia [12], January to April and July to September in India [19], and February to March and July to August in the Bay of Bengal [8]. In Thailand, Sritakon et al. [20] found that this fish spawned throughout the whole year with three peak periods. Nevertheless, none of them simultaneously comprehensively investigated the reproductive trait from the wild population. Moreover, it is known that sex change is an evolutionary adaptation of organisms to enhance a successful reproduction [21, 22]. *E. tetradactylum* is a protandrous hermaphrodite fish, beginning as male and later changing to female. The sizes at the first sex change were reported from different areas. For instance, the Ls_50_ was 32.50 cm of total length (TL) from the north-western Australia [7], and 20.8 cm to 46.5 cm FL across the northern Australia of fork length (FL) from the northern Australia [14]. However, no existing data on the size at first sex change from the Southeast-Asian region is currently available. This study was thus conducted to fulfill the gap and provide scientific knowledge on reproductive biology of *E. tetradactylum.* This is considered the first comprehensive investigation on the reproductive traits of this species.

## Materials and methods

### Study area

**Fig. 1:**
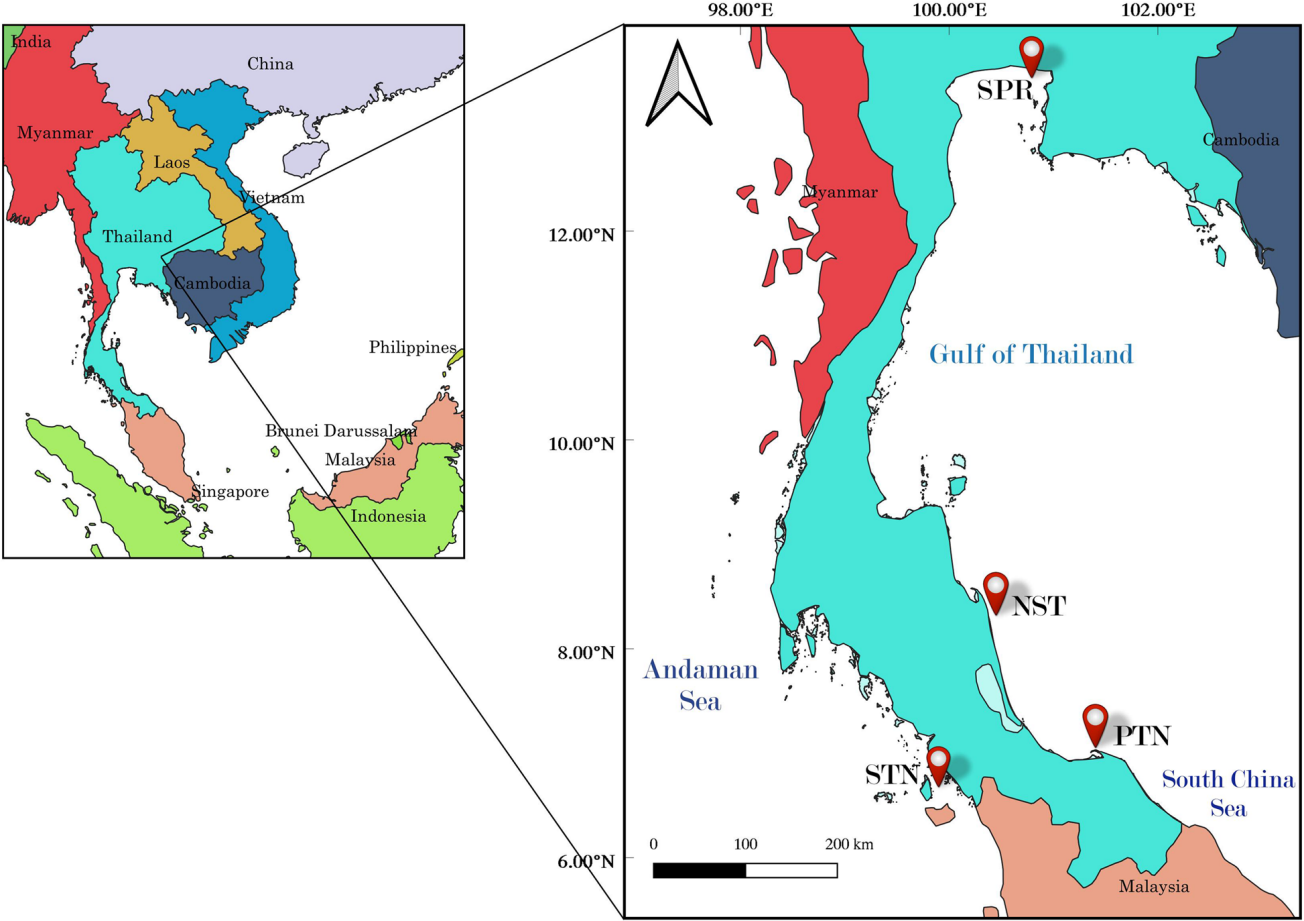
Sampling site of four locations. Sites marked as PTN, STN, NST and SPR represent Pattani Bay, Satun, Nakhon Sri Thammarat and Samut Prakan provinces

The peninsular of Thailand is situated in between the Andaman Sea on the west side and the Gulf of Thailand on the east side. Both coastal regions provide high diversity of fish and are regarded as important fishing grounds [23–25]. The Gulf of Thailand in this study can be divided into two parts: the inner Gulf of Thailand and the southern Gulf of Thailand. The former is mainly influenced by river runoff providing high diversity of fish while the latter is influenced by intrusion of the South China Sea. Four different study sites were selected from both the Gulf of Thailand and Andaman coasts, including the provinces of Pattani (PTN), Nakhon Sri Thammarat (NST) and Samut Prakan (SPR) and Satun (STN) (Fig. 1). Study area was illustrated by QGIS software, version 3.32.1 [26]. Satun province is situated in the coast of Andaman Sea, while Samut Prakan is in the inner Gulf of Thailand, and Nakhon Sri Thammarat and Pattani are in the southern Gulf of Thailand. Among these, Pattani Bay situated in Pattani province is selected as the main study site since it is known as a major fishing ground by local fishermen especially for four-finger threadfin [9, 27, 28], mackerel fish [29–31], shrimps [32] and crabs [33]. Three different seasons were classified in the bay: dry or pre-monsoon from January to April, moderate rainy season (SW monsoon) during May to September, and heavy rainy season or NE monsoon season during October to December [34].

### Data collection

Sampling was conducted monthly from January to December 2021 by a traditional monofilament gill net (mesh size of 4.5 cm stretched, 5 m deep, and 540 m long) in Pattani Bay. The nets were set and left floating for 60 min before being hauled onboard. The sampling period was conducted between 0500–0900 h. The captured specimens, which died immediately after capture, were kept in the ice box and transported to the laboratory at the Faculty of Science and Technology, Prince of Songkla University, Thailand. Approximately 30 specimens of *E. tetradactylum* were selected from a monthly sample for further analysis. Additionally, fish samples from the provinces of Satun (STN), Nakhon Sri Thammarat (NST) and Samut Prakan (SPR) were collected from the catches of local fishermen (Fig. 1).

### Laboratory analysis

Standard length (SL) and body weight (BW) were measured to the nearest 0.01 cm and weighted to 0.01 g, respectively. Consequently, fish samples were later categorized into four size classes: small (18.13 ± 1.71 cm), medium (25.74 ± 2.19 cm), large (32.96 ± 2.06 cm) and extra-large (42.07 ± 2.20 cm) based on the length frequency distribution measured by Past software [35].

A total of 408 fish samples, excluding 21 transitional sex fishes, from Pattani Bay were used to assess the sex ratio, gonadosomatic index (GSI), maturity stage, spawning period and fecundity. The length and weight at first sex change (Ls_50_ and Lw_50_) were calculated for fishes from Pattani Bay (*n* = 429) and Samut Prakan province (*n* = 39), whereas the length at first maturity (Lm_50_) of male and female fish (*n* = 721) were compared for all four sites (Table 1).

**Table 1:** Number of *E. tetradactylum* samples collected from all sites across Thailand during January and December 2021

Season	Month	PTN			SPR			STN			NST			Total
		M	F	T	M	F	T	M	F	T	M	F	T	
Dry	Jan-21	24	7	-	-	-	-	-	-	-	-	-	-	31
	Feb	25	4	3	-	-	-	-	-	-	-	-	-	32
	Mar	16	15	2	-	-	-	-	-	-	-	-	-	33
	Apr	24	5	2	-	-	-	-	-	-	-	-	-	31
SW monsoon	May	35	13	1	-	-	-	-	-	-	-	-	-	49
	Jun	15	12	3	8	23	-	-	-	-	-	-	-	83
	Jul	26	20	5	-	-	-	19	3	-	-	-	-	51
	Aug	14	36	-	-	-	-	-	-	-	-	-	-	50
	Sep	15	13	3	-	-	-	-	-	-	-	-	-	31
NE monsoon	Oct	16	14	-	2	6	-	-	-	-	29	2	-	69
	Nov	16	12	2	-	-	-	-	-	-	196	3	1	230
	Dec-21	16	15	-	-	-	-	-	-	-	-	-	-	31
Total		242	166	21	10	29	-	19	13	-	225	5	1	721

*PTN* Pattani, *SPR* Samut Prakan, *STN* Satun, *NST* Nakhon Sri Thammarat, *M* male, *F* female, *T* transitional sex

### Macroscopic analysis

After removing the gonad from the body cavity, sex and maturity stage of fish samples were distinguished macroscopically with the adapted criteria proposed by Laevastu [36]. It was assigned as male, female or transitional sex based on the presence of both testis and ovary with the relevant literatures [7, 13]. For transitional gonads, the testicular tissue appeared along the dorsal edge and in the inner lateral part of the gonads while the ovarian tissues were along the ventral side and outer part of the gonads. Characteristics of gonadal stages for male and female and transitional sex are given in Tables 2 and 3.

**Table 2:** Characteristic of gonadal development stages for male and female

Stage	Male	Female
Stage I (Immature)	Testes are very small with grey color	Ovaries are small and transparent with yellowish-orange color
Stage II (Resting)	Testes become white, like ribbon	Oocytes in ovaries are still invisible in gonad ovarian wall
Stage III (Developing)	Testes are white and settle approximately half-length of body cavity	Ovaries are larger than stage II and oocytes are visible in gonad ovarian wall
Stage IV (Maturing)	No milt appears and testes occupy more than half-length of body cavity	The ovaries are larger than stage III, creamy orange in color; large oocytes are present in ovarian wall
Stage V (Mature)	Milt appears and 1/3 gonads fill to ventral cavity of testes	Ovaries are massive, yellowish color and occupy approximately 1/2 to 2/3 of body cavity
Stage VI (Spawning)	Milt excludes from testes firm strain to abdominal part	The extensive capillaries of ovary are clearly visible in ovarian wall
Stage VII (Spent)	Testes are smaller than stage V and VI with frail testes not fully empty	Ovaries are smaller than stage V and VI but flaccid and reddish color; some massive oocytes still appear in ovarian wall
Stage VIII (Recovering)	Testes are placid and small with reddish and brown color	Ovaries are small, flaccid and red-dark in color

**Table 3:** Characteristic of gonadal development stages for transitional sex

Stage	Transitional sex
Stage I (Early immature)	Gonads are white, identical to typical male gonad. The testicular and ovarian tissue are separated
Stage II (Late immature)	Gonads become pinkish-white and appear the typical ovary characteristics

### Microscopic analysis

For histological examination, 95 gonads, accounting for 22% of total fish samples, were wiped with tissue paper and weighed to the nearest 0.01 g. They were immediately fixed with 10% buffered formalin and stored in bottle jars [37–39]. Immature individuals for which sex could not be identified with the naked eyes were excluded for this histological analysis. In the histological process, three sub-samples from gonad tissues were taken from three parts of the gonad: anterior, middle, and posterior, respectively. The protocol for further analysis was based on the methodologies described by Zamidi et al. [12], Shihab et al. [13] and Simon et al. [40]. Gonad tissues were loaded in an automatic tissue processor (LEICA JUNG HISTOKINETTE 2000) with the process of 12 solutions consisting of propanol, chloroform and paraplast for 24 h. After cleaning and dehydrating processes, gonad tissues were embedded in the paraffin wax, shaped into blocks and trimmed. The tissues were then transversely sectioned into 4–5 µm slices by a semi-automatic microtome (LEICA Mod. 2035 Biocut). Those tissues were later defiled with hematoxylin and eosin, mounted on glass slides for light microscopy analysis. Finally, gonadal maturation was classified into eight developmental phases viz., stage I (immature), stage II (resting), stage III (developing), stage IV (maturing), stage V (mature), stage VI (spawning), stage VII (spent) and stage VIII (recovering) as indicated by Pember et al. [7], and Shihab et al. [13].

The terminology applied in this study was based on the criteria used by Pember et al. [7], Wallace et al. [41] and Brown‐Peterson et al. [42]. In male, oocyte stages included connective tissue (ct), lumen (l), spermatocytes (sc), sperm duct (sd), spermatogonia (sg), sperm sinuses (ss), spermatids (st), spermatozoa (sz). In transitional gonads, they were connective tissue (ct), nucleolar oocytes (cn), perinucleolar oocytes (pn) and spermatozoa (sz). In female, they were atretic oocytes (ao), cortical alveolar oocytes (ca), nucleolar oocytes (cn), connective tissue (ct), lipid droplets (ld), ovigerous lamellae (ol), primary growth oocytes (pg), post ovary follicle (pof), perinucleolar oocytes (pn), and yolk granule oocytes (yg).

### Data analysis

Sex ratio was calculated by the formula of the total number of females divided by males [43]. The chi-square $({\chi }^{2})$ was later used to testify the monthly difference of sex ratio [44].

To define a monthly reproductive phase of fish, a gonadosomatic index (GSI) of male and female was calculated with the following equation [45]:

$$\mathrm{GSI}=\frac{\mathrm{GW}}{(\mathrm{TW}-\mathrm{GW})}\times 100$$

where GSI is gonadosomatic index, GW is gonad weight (g) and TW is total weight (g). A non-parametric one-way ANOVA (Kruskal–Wallis test) was performed to examine the homogeneity of the monthly means of GSI and number of mature fish using the R program [46].

To determine spawning or breeding season, seasonal variation of percentage of male and female at different gonadal stages was classified as immature (stage I-IV) and mature (stage V-VIII) based on visual observation. The stages of V and above were grouped into mature fish as their readiness to spawn. A spawning season was determined by a high percentage of mature fish [47].

To determine fish fecundity, the posterior, middle and anterior parts of mature female gonads from preserved samples were gently dissected. Eggs were carefully put in a petri dish, weighed to the nearest 0.001 g, and poured with distilled water. The number of eggs was later counted by gravimetric method based on the weight of ovary and density of egg [48]. A fecundity was estimated by a gravimetric method as:

$$\mathrm{F}=({\mathrm{n}}^{*}\mathrm{OW})/\mathrm{WS}$$

where, n = number of eggs in three subsamples, OW = total weight of ovary (g) and WS = weight of three subsample (g). Egg diameter was measured to the nearest 0.1 µm by microscope with the assistance of Digimizer image analysis software version 4.3.4. Then, variances of fecundity among fish size and region were examined. Fish samples from Satun province (STN) were excluded due to an absence of developing/mature ovaries.

Relationship between fecundity (F) and standard length (SL), body weight (BW), gonad weight (GW) and egg diameter (ED) were tested by linear regression analysis [49]. Prior to the analysis, raw data were transformed to Log (x + 1) to reduce non-normality. Then, relationship between fecundity (Y) and independent variables (X) were analyzed with PAST software [35] by the following equation:

$$\mathrm{ln}\left(\mathrm{F}\right)=\mathrm{lna}+\mathrm{b }(\mathrm{lnX})$$

where, a and b are the constant values from linear regression, F is fecundity and X is independent variables (SL, BW, GW and ED). Linear relationship of egg diameter (ED) to SL, BW, GW and fecundity were assessed.

To determine length at the first sex change (Ls_50_), a logistic regression was fitted to the proportions of both male and female in each of the 1 cm length class [50], then applied with PAST software [35] by the following function [51]:

$${\mathrm{P}}_{\mathrm{ls}}=(1+{exp}^{-\mathrm{ln}19 (\mathrm{S}-{\mathrm{S}}_{50})/{\mathrm{S}}_{95}-{\mathrm{S}}_{50})}{)}^{-1}$$

where, *P*_*ls*_ is the proportion of male and female in each interval class *S, S*_*50*_ and *S*_*95*_ are the lengths at which 50% and 95% of the populations are females, respectively. In addition, a similar calculation was applied for weight at the first sex change (Lw_50_) in each of the 10 g of weight class. Due to insufficient samples, fish from STN and NST provinces were excluded for this analysis.

To estimate length at the 50% of fish maturity (Lm_50_**)**, frequency distribution and proportion of both male and female were binned into the 1 cm length class [50]. They were then analyzed by the following logistic function [51]:

$${P}_{lm}=(1+{exp}^{-ln19 (M-{M}_{50})/({M}_{95}-{M}_{50})}{)}^{-1}$$

where, *P*_*lm*_ is the proportion of mature fish in each 1 cm length class *M*, *M*_*50*_ and *M*_*95*_ are the length at which 50% and 95% of the populations are matured, respectively. Due to insufficient samples of female fish from STN and NST, the analysis of Lm_50_ of females for those sites were excluded.

## Results

### Size and sex

Based on the examined samples, the smallest fish was 11.96 cm SL and 36.86 g BW, and the largest was 48.30 cm SL and 1900 g BW. The majority of fish standard length ranged between 20–30 cm SL, accounting for 49.51% of all samples (Table 4). Sex could be allocated into three categories viz., male, female and transitional sex. The average SL and BW of males, females and transitional sex were 22.36 ± 4.96 cm, 30.43 ± 5.29 cm and 28.49 ± 3.68 cm, and 229.69 ± 168.90 g, 520.92 ± 265.47 g and 436.91 ± 168.54 g, respectively. Spatially, the lowest SL (19.38 ± 2.86 cm) and BW (137.51 ± 67 g) were found in NST, while the highest SL (30.02 ± 4.86 cm) and BW (442.76 ± 205.4 g) were in SPR.

**Table 4:** Size attributes of *E. tetradactylum* collected from coastal areas of Thailand

Size class	Min	Max	Number (N)	95% CI	Standard length (mean SL ± SD)	Weight of fish (mean BW ± SD)
Small	11.96	21.04	237	17.91 − 18.35	18.13 ± 1.71	110.59 ± 29.67
Medium	21.13	29.89	357	25.51 − 25.97	25.74 ± 2.19	316.43 ± 89.11
Large	30.13	38.61	103	32.58 − 33.39	32.96 ± 2.06	614.70 ± 151.83
Extra Large	39.25	48.30	24	41.12 − 43.02	42.07 ± 2.20	1116.47 ± 261.12
Sex						
Male	11.96	44.32	496	21.29 − 22.80	22.36 ± 4.96	229.69 ± 168.90
Transitional sex	22.07	37.57	22	26.82 − 30.16	28.49 ± 3.68	436.91 ± 168.54
Female	22.37	48.30	203	29.69 − 31.16	30.43 ± 5.29	520.92 ± 265.47
Site						
STN	19.83	28.26	22	21.85 − 23.90	22.87 ± 2.25	215.25 ± 64.93
PTN	11.96	48.30	429	26.83 − 27.92	27.37 ± 5.75	409.13 ± 250.33
SPR	22.52	42.34	39	28.42 − 31.62	30.02 ± 4.86	442.76 ± 205.40
NST	14.76	31.55	231	19.01 − 19.75	19.38 ± 2.86	137.51 ± 67

*STN* Satun, *PTN* Pattani, *SPR* Samut Prakan, *NST* Nakhon Sri Thammarat

The overall sex ratio between male and female was 1:0.69 (χ^2^ = 14.16, *p* < 0.001) (Table 5). Monthly sex ratio varied sporadically, including in January, February, April, May and August (χ^2^ > 3.84, *p* < 0.05).

**Table 5:** Monthly sex ratio of *E. tetradactylum* collected in Pattani Bay from January to December 2021

Season	Month	Male	Female	Transitional sex	Sex ratio (M:F)	${\mathrm{\rm X}}^{2}$	*P* Value
Dry	Jan-21	24	7	-	1:0.29	9.32	**0.002**
	Feb	25	4	3	1:0.16	15.21	** < 0.001**
	Mar	16	15	2	1:0.93	0.03	0.86
	Apr	24	5	2	1:0.21	12.45	** < 0.001**
SW monsoon	May	35	13	1	1:0.37	10.08	**0.001**
	Jun	15	12	3	1:0.8	0.33	0.56
	Jul	26	20	5	1:0.77	0.78	0.38
	Aug	14	36	-	1:2.57	9.68	**0.002**
	Sep	15	13	3	1:0.86	0.14	0.71
NE monsoon	Oct	16	14	-	1:0.87	0.13	0.72
	Nov	16	12	2	1:0.75	0.57	0.45
	Dec-21	16	15	-	1:0.93	0.32	0.86
Total		242	166	21	1:0.69	14.16*	** < 0.001**

^*^ $${(\upchi }^{2}, p<0.01)$$, (Critical value $${(\upchi }^{2}$$_0.95_ = 3.84)

### Gonadosomatic index (GSI) and maturity

**Fig. 2:**
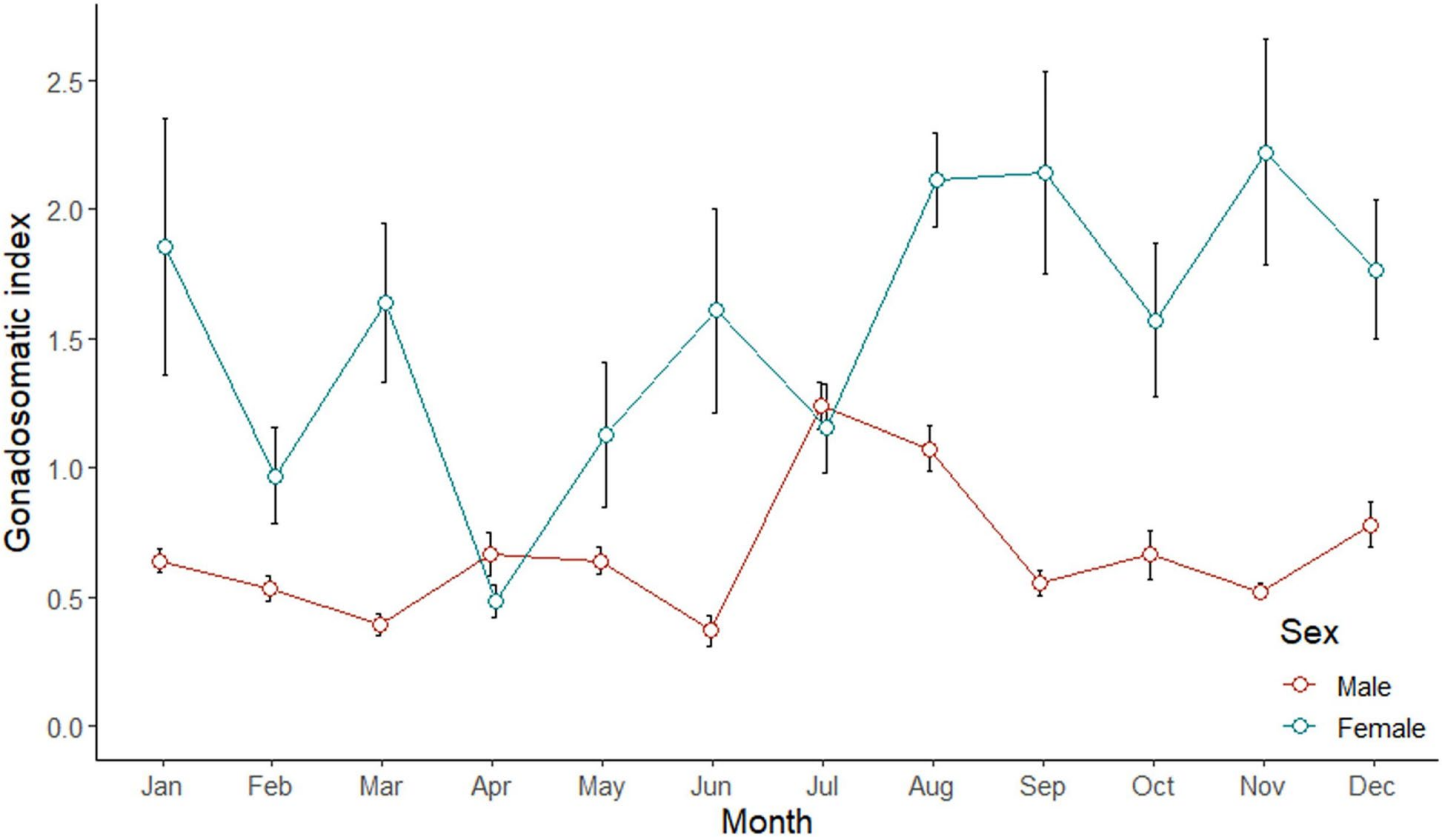
Monthly variation of gonadosomatic index (mean ± SE) of male and female *E. tetradactylum* collected from Pattani Bay during January and December 2021

**Fig. 3:**
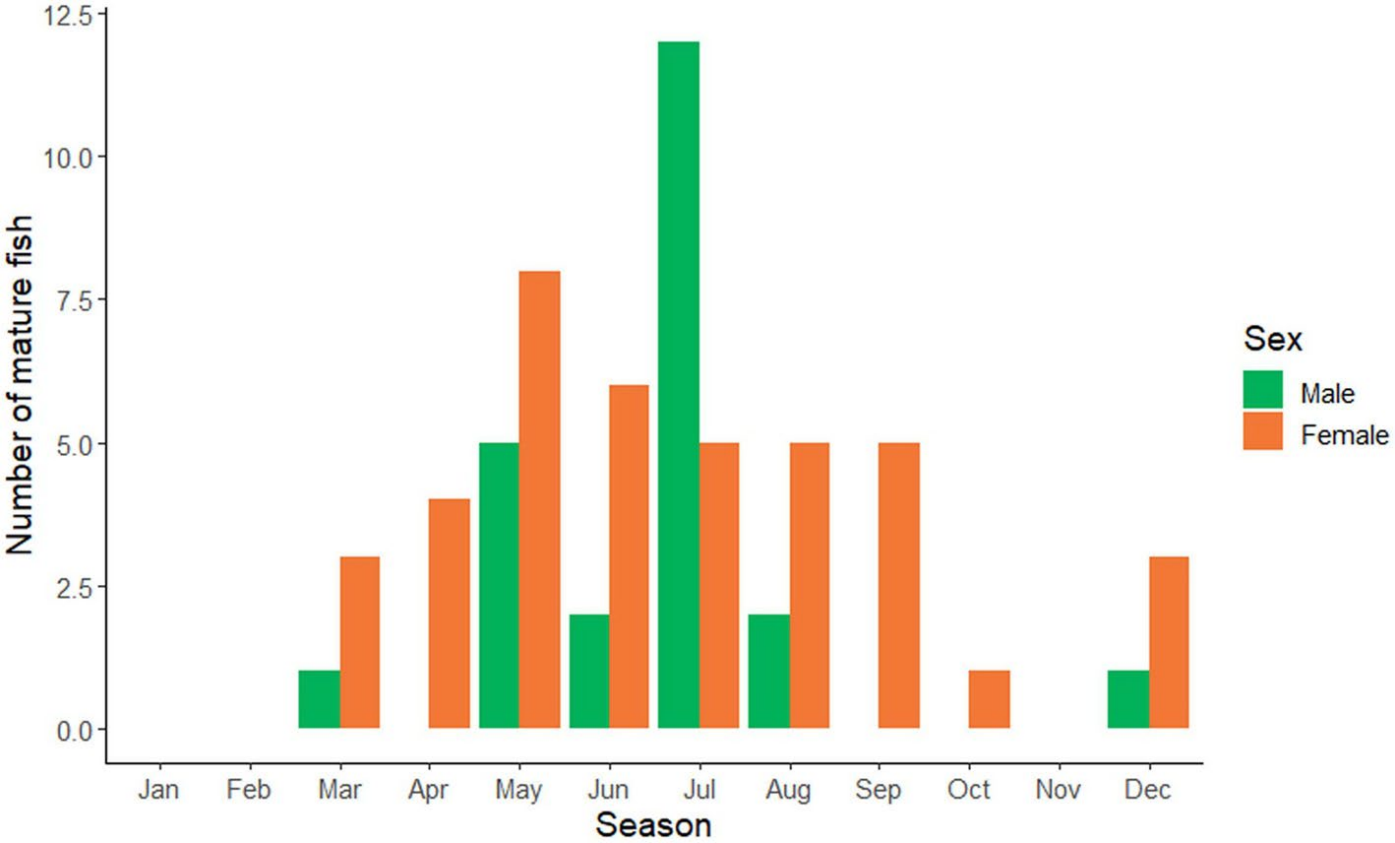
Number of mature males (*n* = 242) and females (*n* = 166) of *E. tetradactylum* collected in Pattani Bay during January and December 2021

The Kruskal–Wallis test indicated different GSI values among months (*p* < 0.05). Different patterns of mean GSI between male and female were observed (Fig. 2). The GSI of males was the highest in July and dropped gradually from September onwards. The GSI of females demonstrated five times of peak within a year, indicating multiple spawning behavior. The highest number of mature males was observed in July while mature females in May (Fig. 3). It coincided with the peak of male’s GSI which was observed in July as the observation of high preponderance of mature male. Kruskal–Wallis test indicated that there was no variation between the number of mature males and females among months (*p* > 0.05).

**Fig. 4:**
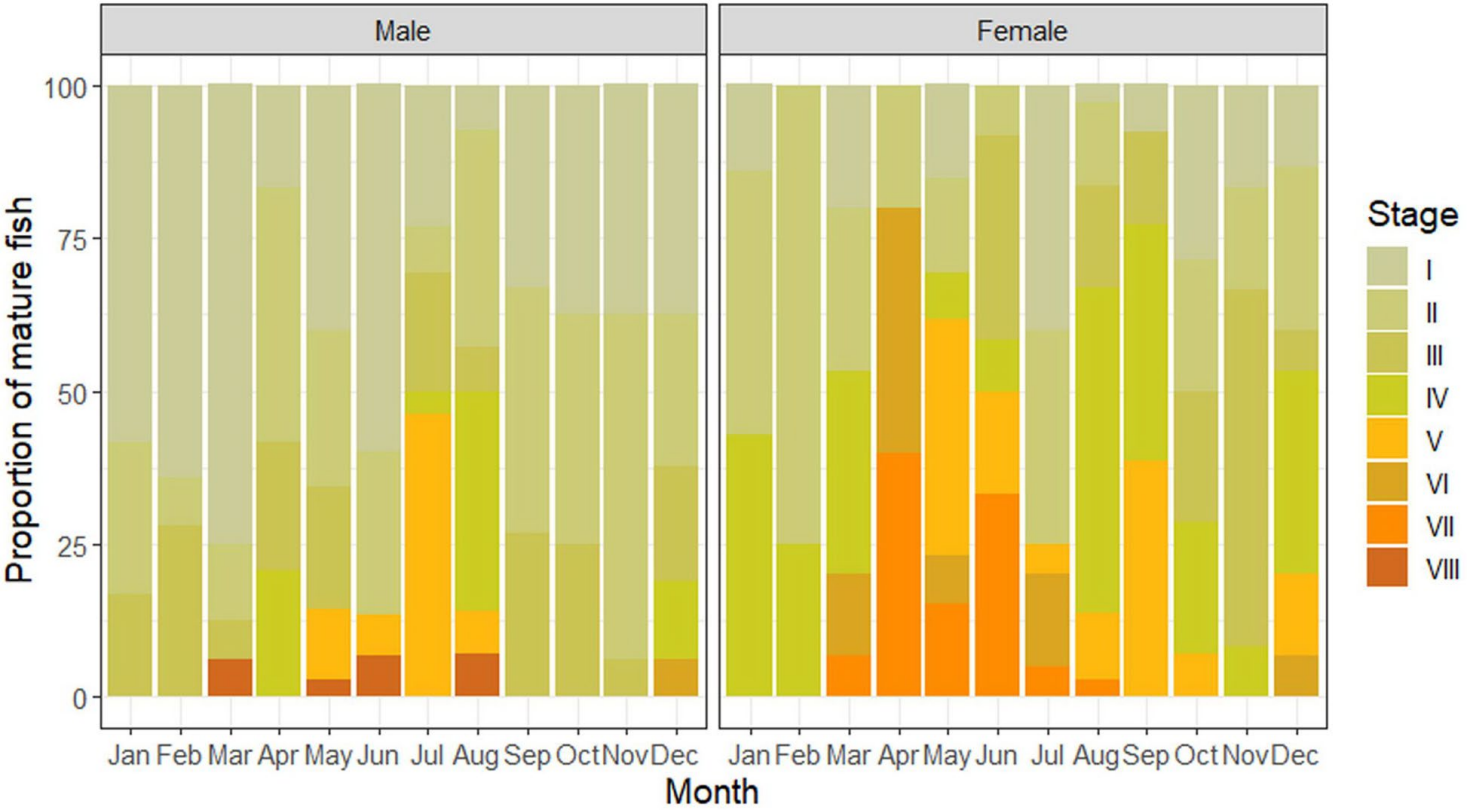
Percentage of seasonally frequencies of males (*n* = 242) and females (*n* = 166) maturity stages of *E. tetradactylum* collected in Pattani Bay during January and December 2021

There were eight maturity stages observed in males but no resting stage (Stage II) found in females during the period of this study (Fig. 3). Overall, 91.62% of males and 73.66% of females were found to be from immature to maturing stages throughout the year. The highest percentage of mature males (46.15%) and females (80%) occurred in July and April, respectively (Fig. 4). Occurrence of mature stages of females (Stage V-VIII) during May to October and December indicated that this fish spawned throughout the whole year.

### Stage of gonad maturity

**Fig. 5:**
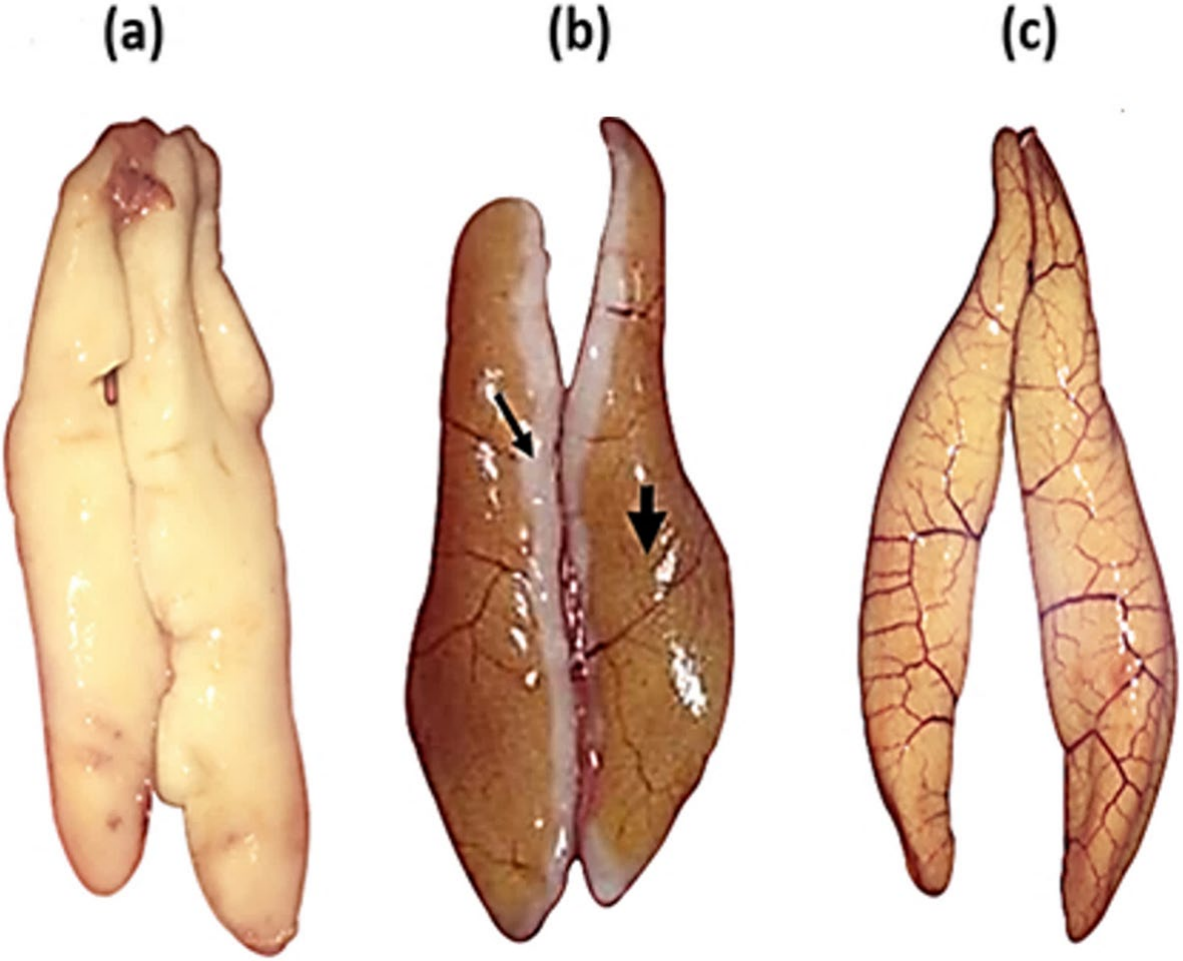
Macroscopic pictures of gonads. **a** Testis, **b** transitional gonad and **c** ovarian. The small arrow indicates testicular tissue, and the larger arrow indicates ovarian tissue of transitional sex of *E. tetradactylum*

**Fig. 6:**
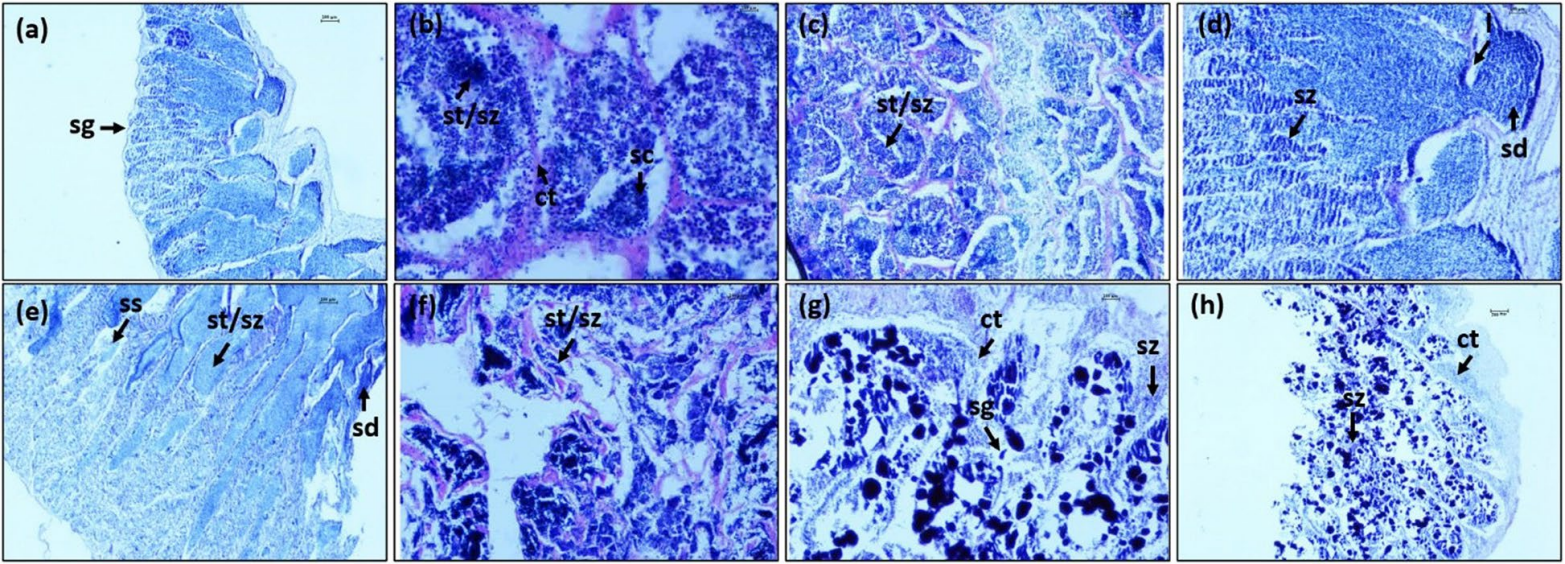
Histological section of male gonads. **a** immature, **b** resting, **c** developing, **d** maturing, **e** mature, **f** spawning, **g** spent and **h** recovering stages of *E. tetradactylum*. ct: connective tissue, l: lumen, sc: spermatocytes, sd: sperm duct, sg: spermatogonia, ss: sperm sinuses, st: spermatids, sz: spermatozoa

Gonad maturation could be categorized into male (Fig. 5a), transitional sex (Fig. 5b) and female (Fig. 5c). The standard length of fish ranged from 11.96—44.32 cm SL in male, 22.07—37.57 cm in transitional sex and 22.37—48.30 cm in female. Histological analysis indicated that the presence of spermatogonia (sg) predominantly occurred in immature male gonads (Fig. 6a). It was distinguished by a slightly stained cytoplasm and homogenous nuclei. The connective tissue (ct), spermatocyte (sc), spermatids (st) and spermatozoa (sz) appeared in the resting, developing and maturing stages (Fig. 6b, c and d, respectively). These stages represented an active spermatogenesis, and a number of spermatozoa could be recognized. The presence of lumen (l) in male gonads appeared in the maturing stage (Fig. 6d). When *E. tetradactylum* became mature, other characteristics including sperm sinuses (ss) and sperm duct (sd) appeared in the gonad wall (Fig. 6e). Sperm duct and majority of the testes were full of spermatids (st) and spermatozoa (sz). The presence of crypts containing spermatozoa (sz) was usually captive in the outer perimeter of testis in the spawning stage (Fig. 6f). The largest numbers of spermatogonia (sg) and spermatozoa (sz) were found in the spent stage.

**Fig. 7:**
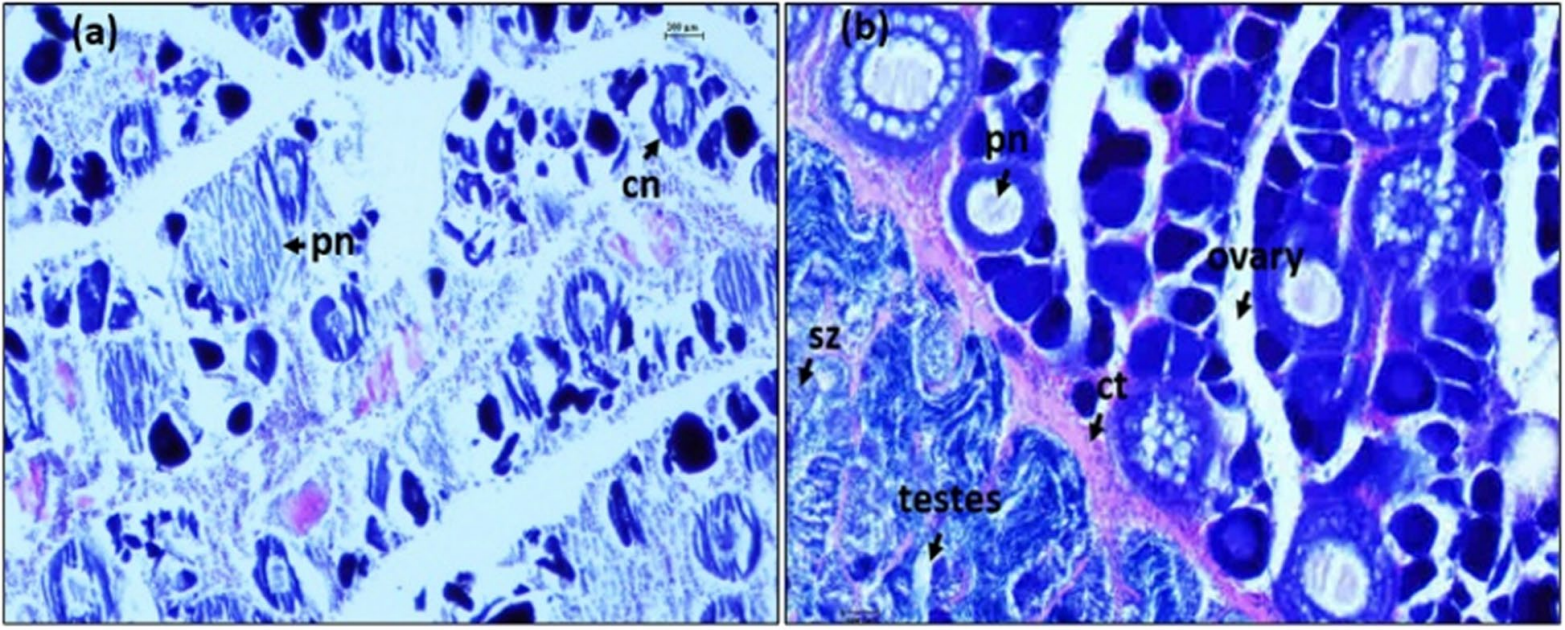
Histological section of gonad. **a** early transitional and **b** late transitional stage of *E. tetradactylum*. cn: chromatin nucleolar, ct: connective tissue, pn: perinucleolar oocytes, sz: spermatozoa

**Fig. 8:**
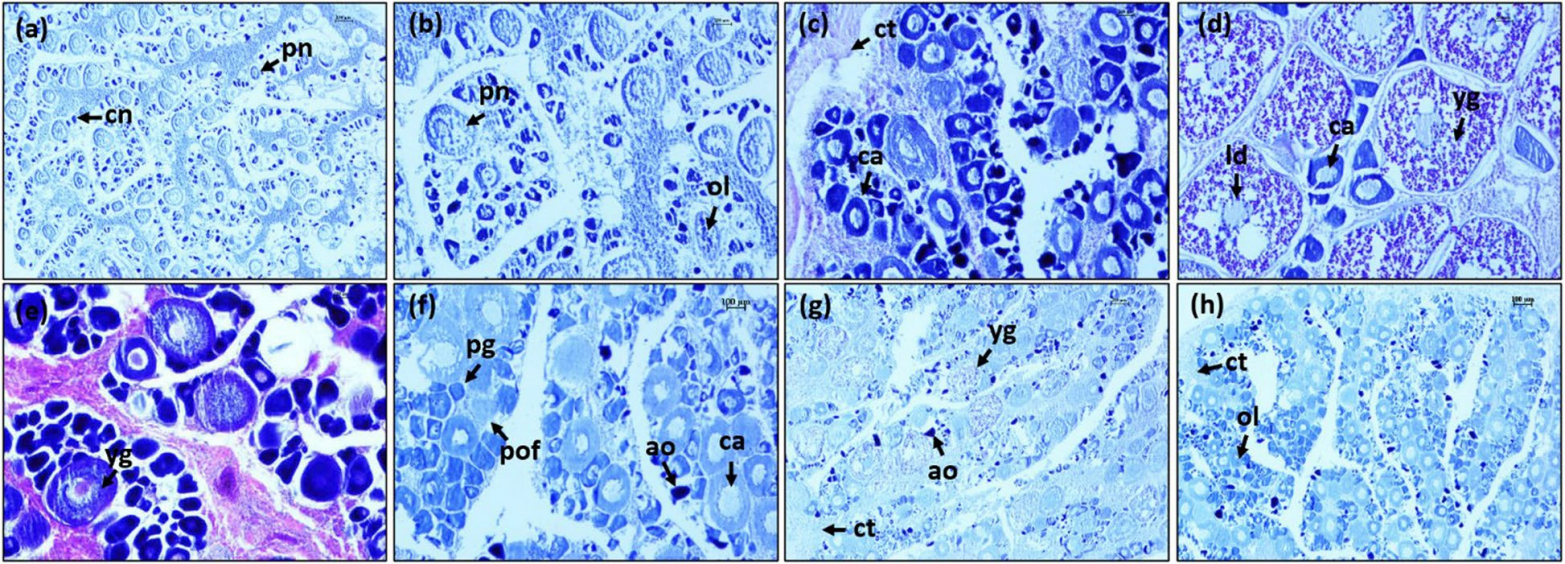
Histological section of female gonads. **a**-**b** immature, **c** developing, **d** maturing, **e** mature, **f** spawning, **g** spent and **h** resting of *E. tetradactylum*. ao: atretic oocytes, ca: cortical alveolar oocytes, cn: nucleolar oocytes, ct: connective tissue, ld: lipid droplets, ol: ovigerous lamellae, pg: primary growth oocytes, pn: perinucleolar oocytes, pof: post ovulatory follicle, yg: yolk granule oocytes

However, the spaces of empty sperm were observed within the connective tissue (Fig. 6g). In the recovering stage, testes are dominated by connective tissues (ct) consisting of extensive spaces with a few appearances of spermatozoa (sz) (Fig. 6h). This appearance of spermatozoa indicated an undergoing spermiation of the gonad. In transitional gonads, the testicular and ovarian tissues were separated by the connective tissue (ct). The presence of chromatin nucleolar oocyte (cn) and perinucleolar oocytes (pn) in early transitional gonads occurred with an interseptum of connective tissues (Fig. 7a). While in the late transitional period, size of testicular region was reduced and gonad assumed as a typical characteristic of ovaries with large numbers of perinucleolar oocytes (pn) (Fig. 7b). In female gonads, chromatin nucleolar oocyte (cn), perinucleolar oocytes (pn) and ovigerous lamellae (ol) are present in immature ovaries with small nucleoli (Fig. 8a and b). The cytoplasm was strongly basophilic in this stage. During the ovary developing stage, cortical alveolar oocytes (ca) presented clearly (Fig. 8c) and the ovaries began to develop but were not ready to spawn. Maturing and mature stages were notable by the presence of cortical alveolar (ca) and abundance of yolk granule oocytes (yg) with several lipid droplets (ld). The number of yolk granule oocytes (yg) were large (Fig. 8d and e, respectively). Primary growth oocytes (pg), atretic oocytes (ao) and some ripped eggs (rp) were found in the spawning stage (Fig. 8f). Remnant yolk granule oocytes (yg) were recorded in the spent stage and atretic oocyte (ao) and connective tissues (ct) were typically detected (Fig. 8g). Connective tissue (ct) and ovigerous lamellae (ol) appeared in the recovering stage (Fig. 8h) indicating the continued process of ovulation.

### Fecundity

**Fig. 9:**
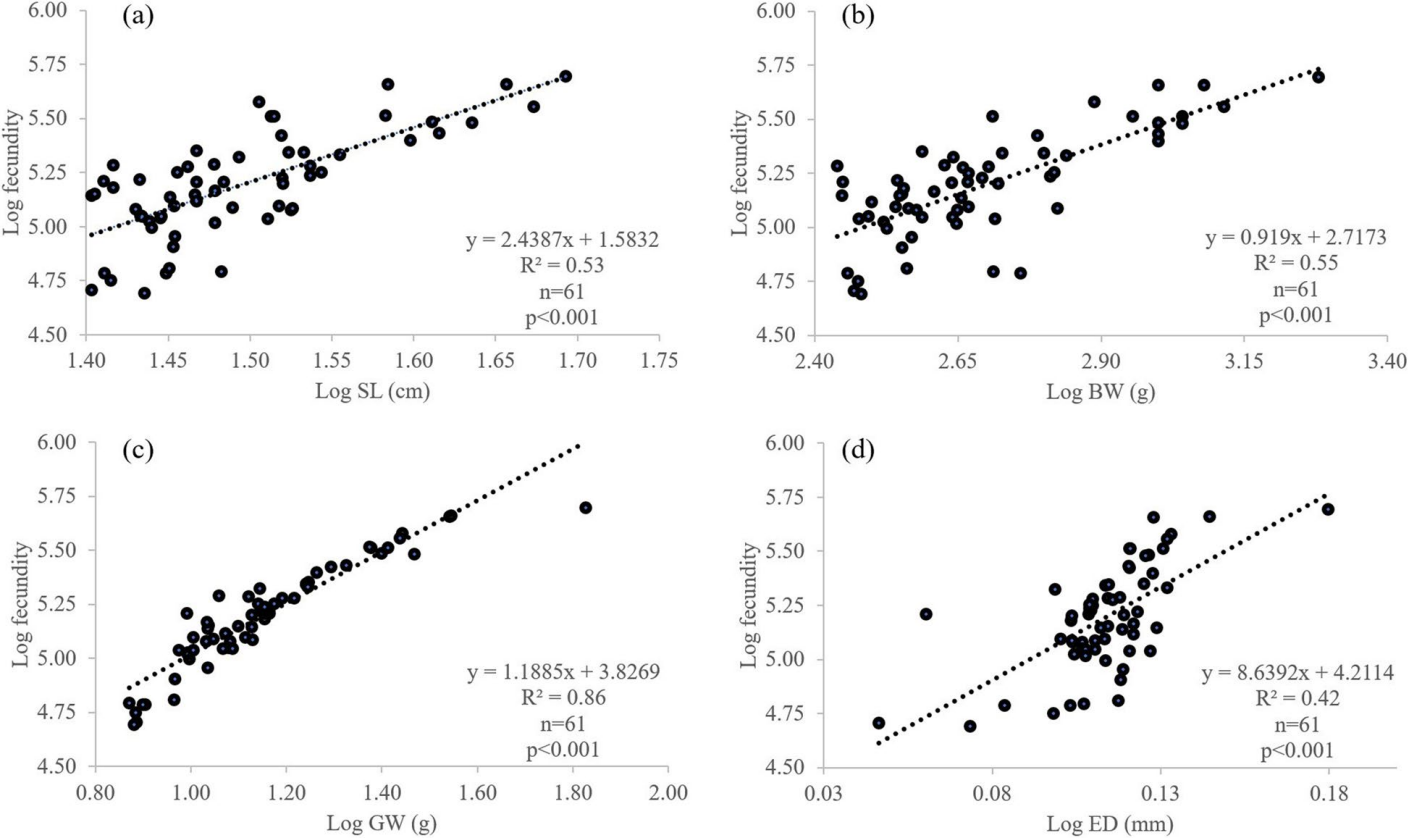
Relationship between fecundity and **a** standard length (SL), **b** body weight (BW), **c** gonad weight (GW) and **d** egg diameter (ED) of *E. tetradactylum* collected from Pattani Bay during January and December 2021

**Fig. 10:**
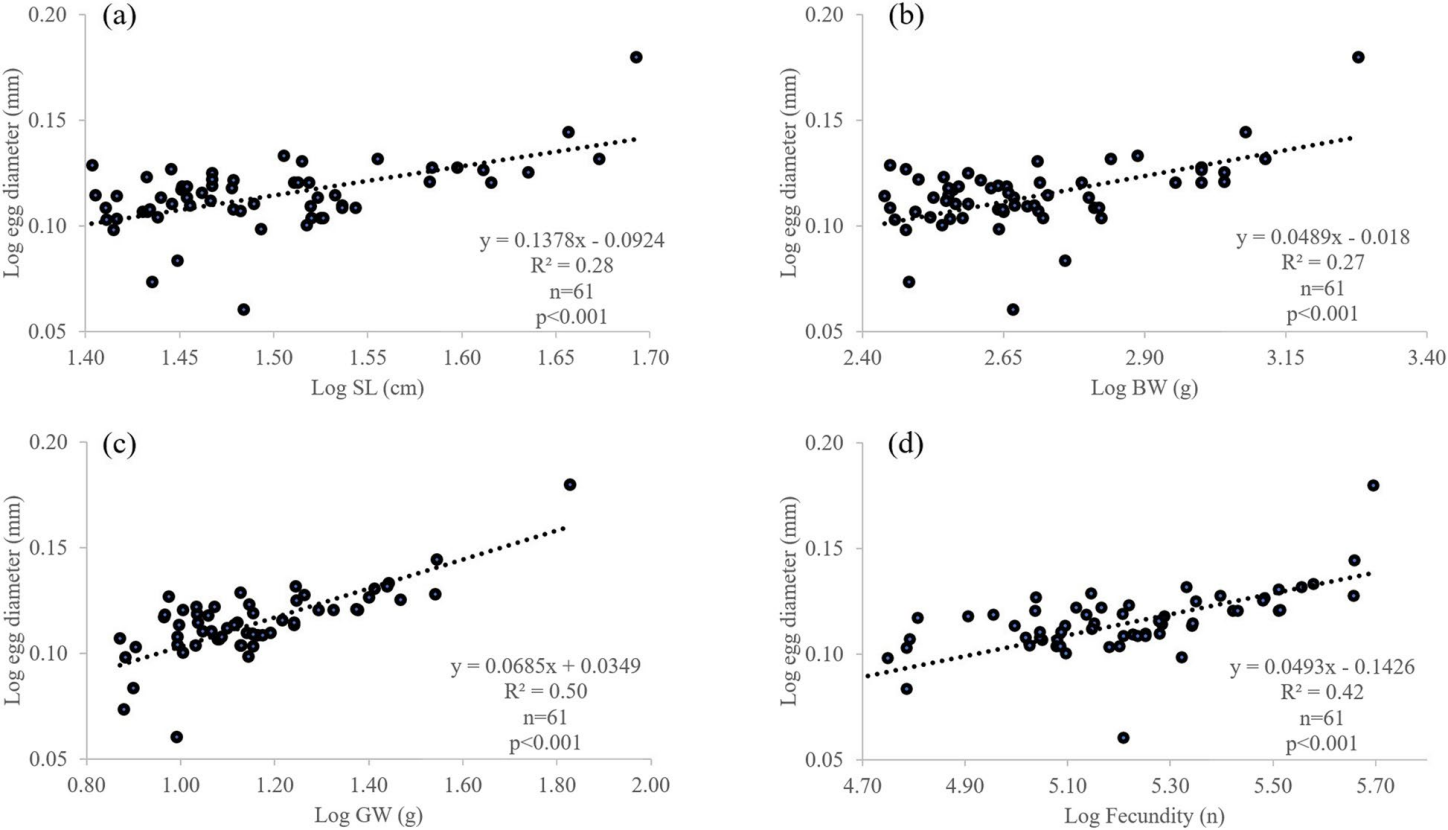
Relationship of fecundity of fish between egg diameter (ED) and body parameters. **a** Standard length (SL), **b** body weight (BW), **c** gonad weight (GW) and **d** Fecundity (F) of *E. tetradactylum* collected from Pattani Bay during January and December 2021

A total of 78 female fishes with developing and maturing ovaries, SL ranging from 22.37 to 48.30 cm, were estimated for fecundity. It was found that the fecundity varied between 0.49 × 10^5^ to 4.96 × 10^5^ eggs, with an average of 1.85 × 10^5^ ± 1.05 × 10^5^ eggs and increased along an increment of fish size (Table 6). The relationship between fish fecundity and egg diameter was observed in which the higher fecundity posed the greater egg diameter. Fecundity also differed between study sites (*p* < 0.05) where fish from SPR had the highest compared to fishes from the other two regions, PTN and NST (Table 6). Positive logarithmic relationships of fish fecundity (F) to SL, BW, OW and ED from PTN were observed (Fig. 9). Positive correlations of fecundity to gonad weight (*R*^2^ = 0.86) and ED to fecundity (*R*^2^ = 0.52) were greater than other body parameters (Figs. 9 and 10). Moreover, a positively higher correlation between ED and GW was found compared to other body parameters.

**Table 6:** Fecundity of *E. tetradactylum* collected from coastal waters of Thailand

	N	Standard length (cm)	Weight (g)	Gonad weight (g)	Fecundity Min–Max (× 10^5^ eggs)	Fecundity Mean ± SD (× 10^5^ eggs)	Egg diameter (mm)
Overall	78	22.37–48.30	186.9–1900.0	5.99–66.24	0.49–4.97	1.85 ± 1.05	0.30 ± 0.05
Size class							
Medium	39	22.07–29.86	186.9–573.5	5.99–16.65	0.49–2.24	1.23 ± 0.46	0.11 ± 0.05
Large	33	30.13–38.61	346.9–1100	6.81–38.06	0.72–4.89	2.24 ± 1.02	0.30 ± 0.03
Extra large	6	39.86–48.30	1000–1900	20.12–66.24	2.70–4.96	3.65 ± 0.92	0.37 ± 0.07
Study Site							
PTN	61	24.3–48.30	273.9–1900	6.44–66.24	0.49–4.96	1.81 ± 1.04	0.3 ± 0.05
SPR	15	23.34–37.93	186.9–800	6.81–36.80	0.72–4.89	2.08 ± 1.15	0.3 ± 0.03
NST	2	22.07–31.55	209.4–485	5.99–14.72	0.56–1.78	1.17 ± 0.87	0.27 ± 0.04

*PTN* Pattani, *SPR* Samut Prakan, *NST* Nakhon Sri Thammarat

### Length and weight at first sex change and size at first maturity

**Fig. 11:**
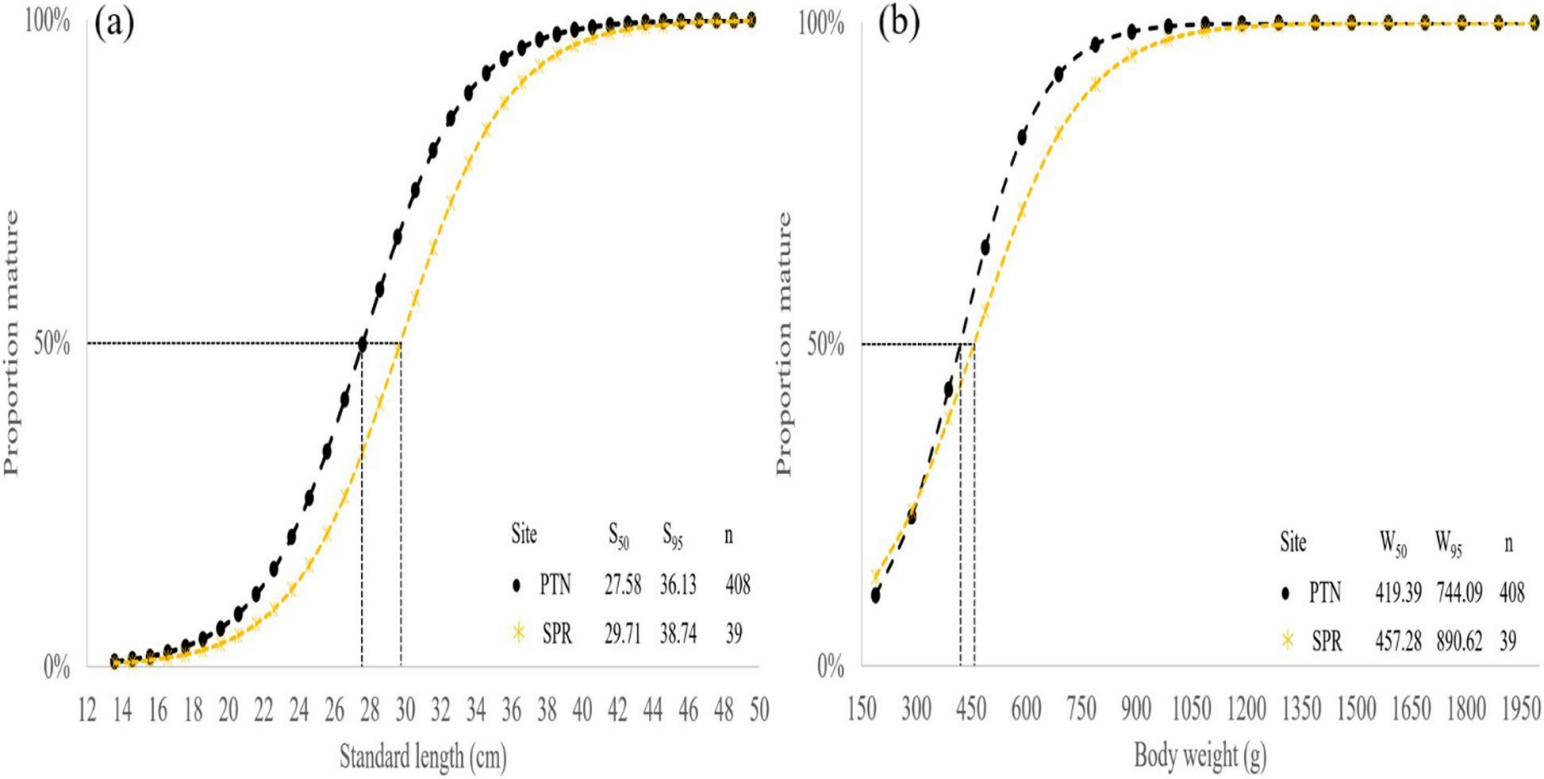
Percentage of length (**a**) and weight (**b**) at the first sex change (L_s50_) of *E. tetradactylum* from Pattani (PTN) and Samut Prakan (SPR). Cut-off 50% indicated fish at the first sex change

**Fig. 12:**
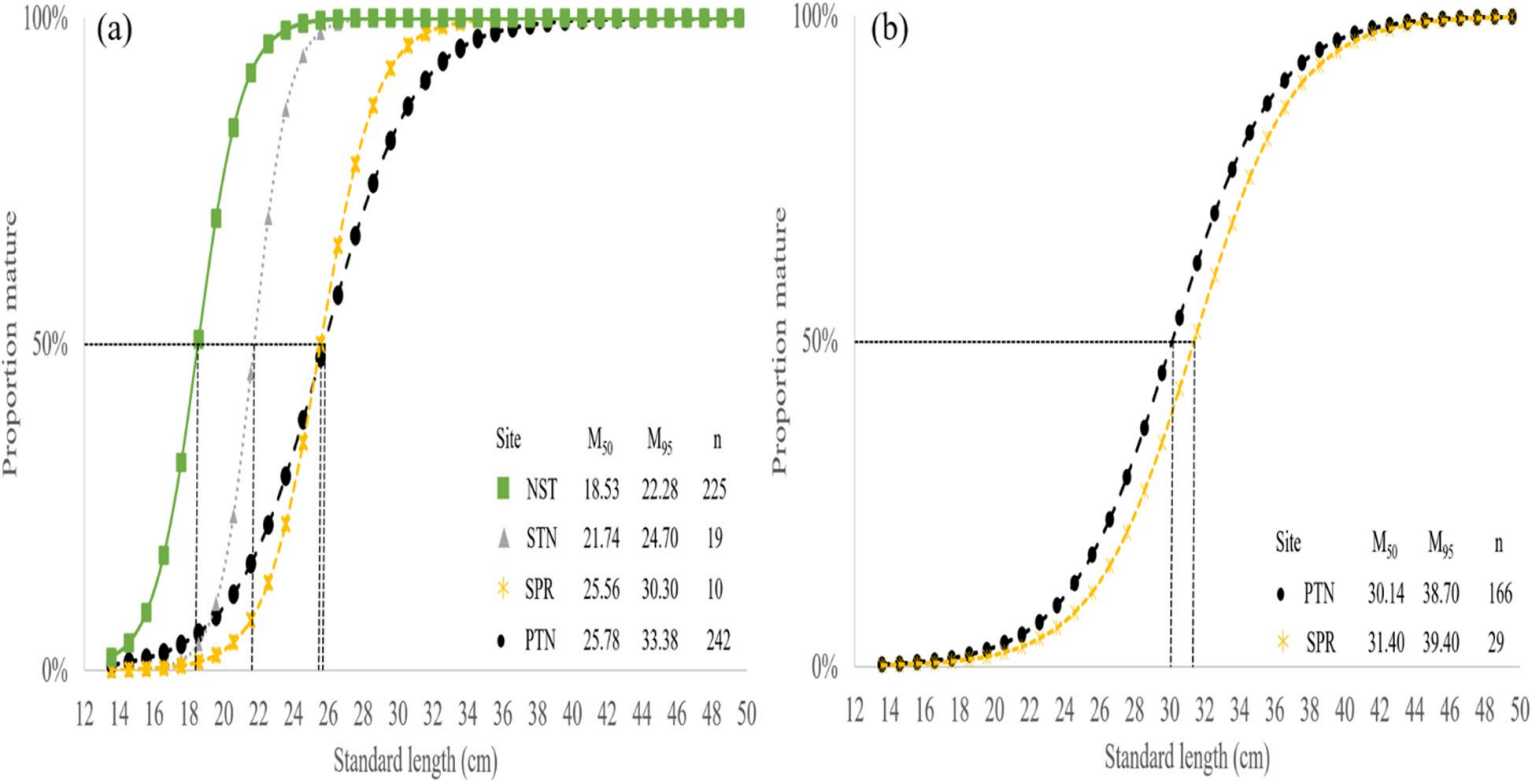
Percentage of mature male (**a**) and female (**b**) of size at first maturity (Lm_50_) of *E. tetradactylum* collected from four sites. Cut-off 50% indicated fish was first mature. NST = Nakhon Sri Thammarat, PTN = Pattani, STN = SATUN and SPR = Samut Prakan

Results from logistic regression on the proportion of males and females indicated variations of Ls_50_ and Lw_50_ from two different regions. The Ls_50_ and Lw_50_ of fish from PTN and SPR were 27.58 cm SL and 419.39 g BW and 29.71 cm SL and 457.28 g BW, respectively (Fig. 11). The Lm_50_ of both males and females were estimated at 18.53 cm SL and 30.14 cm SL, respectively. By comparing among all study sites, results from logistic regressions indicated that Lm_50_ of males from NST matured at 18.52 cm SL, which was earlier than other sites. Female fish from PTN and SPR matured at 30.14 cm SL and 31.40 cm SL, respectively (Fig. 12).

## Discussion

This study provides an intensive aspect on the reproductive traits of *E. tetradactylum* along the coasts of Thailand. It was observed that the majority of fish samples collected by a traditional gill net, a selective fishing gear, were larger than 20.0 cm SL with the maximum size of 48.3 cm SL (Table 4). All fishes found in this study with the size of smaller than 22.0 cm SL are male. Theoretically, the sex ratio between male and female of the population is 1:1, in exception of the organism living under a particular circumstance [52]. Some factors that may influence sex ratio of fish are fishing, mortality, migration, environmental conditions including reproductive behavior [53], fish size classes [54], growth rate [52], different period of maturity [12, 55] and different position in the water column [56]. The variations of sex ratio in different fish species from different ecosystems have been reported widely. For instances, different sex ratio was observed in Lethrinids fish (*Lethrinus miniatus*) [50], puffer fish (*Carinotetraodon travancoricus*) [57] and sawtooth barracuda, *Sphyraena putnamae* [58]. On the contrary, some studies reported a greater proportion of males than females [52] and some highlighted females predominated males [54, 59]. Different migratory patterns between male and female were also reported to influence sex ratio [53, 54, 58]. Moreover, size at first sex change affected sex ratio of the mature fish stock structure [14, 50]. Results from this study indicated that the overall sex ratio between male and female of *E. tetradactylum* from PTN was 1:0.69, which is highly predominated by males especially in January, February, April, May and August (*p* < 0.05). This circumstance, as a greater proportion of male found in dry and SW monsoon seasons (Table 5), is probably because this fish is a protandrous hermaphrodite, which initially grows as male and later changes to female at certain age, thus more male can be found in the coastal habitat where small sized-fish dominates this ecosystem. This is supported by a report from Zamidi et al. [12] who indicated that males fish dominated females from gillnets in Malaysia. Another study from the rearing pond by Cheng et al*.* [60] found that the ratio of females was 34.5% in the first year of culture and changed to 90.3% after three years in the rearing pond. An equal sex ratio in monsoon season might be related to seasonal reproduction and pattern of sex change. Future study on this particular aspect is essential*.*

Gonadosomatic index (GSI) indicates spawning or breeding season of fish [55, 56], as an increment of GSI value posing an increasing reproductive activity [40]. The GSI normally increases along with the maturation of fish at peak during the spawning period and declines rapidly after spawning [52]. This study found that the GSI value of females is higher than males reflecting a greater proportion of females’ bodies retained the larger gonad [52, 53, 56]. The significant monthly variation of mean GSI was found for both male and female (*p* < 0.05). The trend of GSI values found in this study indicates multiple spawning behavior of this fish species. It is related to the greater proportion of mature gonasd found along with high GSI values in an exception of the period without mature females in November. Moreover, the greatest number of mature males and females were also observed in moderate rainy season (SW monsoon) and heavy rainy season (NE monsoon), indicating higher chance to breed and spawn during these periods. Thus, it can be concluded that the spawning season of *E. tetradactylum* in PTN, based on the GSI value, proportion of mature gonad and number of mature fish, occurs the whole year round with the highest period in moderate rainy and heavy rainy seasons. This finding supports some earlier studies reported spawning periods of *E. tetradactylum* from different regions. This fish species was found to have a lengthy spawning period with peak in June in Malaysian coastal waters [12] and peak from October to December in the north-western part of Australia [7]. Those findings also prove that habitat and season influence spawning patterns of fish. Similar to other fish species, environmental conditions during monsoon season are suitable for breeding activity due to strong water current, high turbidity, low temperature, low sunlight [52, 55, 56, 58] and less predation [53]. As spawning strategy and maturity phase of male and female fishes can be determined by the GSI and histological examination of gonads [61, 62], results from gonad maturation indicate the synchronous and asynchronous spawning behavior of male and female, respectively. Male can be defined as a total spawner reproducing once a year, whilst female is an asynchronous fish or a multiple spawner reproducing several times a year [42, 63, 64]. This finding supports the study conducted by Cheng et al. [60] who reported an asynchronous reproductive pattern of this fish from an aquaculture pond in Taiwan.

Fecundity data is able to support an exploitation of fish stock, population dynamics and fisheries management practices [52, 53]. The four-finger threadfin is a benthic-pelagic species which tends to spawn with a small number of eggs but larger in size [42, 55, 64, 65]. The average fecundity from this study is 1.84 × 10^5^ eggs, considerably lower than those reported from other areas. The earlier studies of fecundity from Malaysia, Bay of Bengal and Taiwan were reported at 3.41 × 10^5^—11.14 × 10^5^ eggs [13], 10.05 × 10^5^—20.91 × 10^5^ eggs [8] and 3.17 × 10^5^—25.08 × 10^5^ eggs [60], respectively. However, these earlier reports investigated the bigger size classes of fish compared to the present study, thus the variation of fish size may directly affect the fecundity [56, 58]. This study also confirms that fecundity increased proportionally with an increasing body and gonad weights [12, 40, 66]. Relationships between fecundity and body characteristics were used to assess corresponding among them. More accurate assessment of relationship when gonad weight was used as indicator to correlate with fecundity, instead of length and weight of fish [66]. This study found that egg diameter is positively correlated with gonad weight and fecundity. This pattern of relationship, as the larger fish size corresponding to the heavier gonad and leading to the bigger egg size, was highlighted by Nesarul et al. [8]. However, Thorsen et al. [67] found different results for the Atlantic cod in which fecundity decreased when egg diameter increased. Moreover, the present study also observed that fishes from different sites produce different numbers of fecundities, with the highest at SPR compared to PTN and NST.

Knowledge on sex change during ontogeny is useful for fisheries management practice [22, 68]. Several factors regulate the size at sex change such as body size [22, 49, 68], predation, fishing pressure [14, 68, 69], breeding season [69] and parental care [70]. This study confirms that *E. tetradactylum* is a protandrous hermaphrodite with the length at first sex change (Ls_50_) of 27.58 cm TL and weight (Lw_50_) of 419.39 g. This size is considered smaller compared to the earlier reports from other areas. Thus, locality may affect the size at first sex change of this fish. This is coincident with a report by Shimizu et al. [70] who suggested that the estimated value of Ls_50_ differed across the locality where fishes resided. For instance, it was 32.5 cm TL in the north-western Australia, 40.0 cm TL in the western Australia [8], 20.8 cm to 46.5 cm FL across northern Australia [14]. Pember et al. [7] found that *E. tetradactylum* in eastern Queensland grew up to 100 cm TL at the age of 8 years and sex change at 40 cm TL or equivalent to 2 years. They also suggested that the first sex change of this fish took at least a year to reveal the transitional sex after spawning season. Similarly, the sex change of *Labrus bergylta* occurred at the end of their breeding season [69]. An estimation of the first maturation of fish (Lm_50_) is essential to apply for assessing a minimum permissible mesh size and avoid over exploitation of juveniles for fisheries management [54, 60, 71]. Environmental conditions can lead to changes of growth and onset of gonadal maturation [63, 64, 72]. Stock density and food availability can also influence sexual maturity of marine fish species [58]. Generally, female fish grow larger than male but the male matures earlier than female [52, 57] due to different energy consumption [43]. However, this is not the case for *E. tetradactylum* as it starts the earlier age as male and later changes to female during ontogeny. This study indicates that male and female fishes larger than 25.78 cm SL and 31.40 cm SL, respectively, can be defined as mature. It is slightly different from other areas such as 20.11 cm TL for male from Australia [7], 31.10 cm FL for pooled sex from India [11], and 39.60 cm FL for pooled sex from Indonesia [10]. Hence, this study provides the first report on the Lm_50_ values for both male and female simultaneously.

## Conclusion

The present study illustrates reproductive aspects of *E. tetradactylum* in the coastal waters of Thailand. Male fish predominated the annual stock with the overall sex ratio of 1:0.69. It spawned the whole year round with peaks in moderate rainy and heavy rainy seasons. Histological examination confirms the protandrous hermaphrodite posing multiple spawning habits. The average fecundity was 1.85 × 10^5^ ± 1.05 × 10^5^ and positively related with standard length, body weight, gonad weight and egg diameter. The Ls_50_ and Ws_50_ for fishes from Pattani Bay and Samut Prakan province were 27.58 cm, 419.39 g and 29.71 cm, 457.28 g, respectively. The Lm_50_ of male from Pattani Bay and Samut Prakan province were 25.78 cm and 25.56 cm. This study thus provides crucial scientific information on the reproductive biology of *E. tetradactylum* which is fundamental for the management of natural fish stock and aquaculture development of this species.

## Data Availability

The data that support in this study are accessible on request from the corresponding author.
